# Hyperglycemia Induced in Sprague–Dawley Rats Modulates the Expression of CD36 and CD69 During Wound Healing

**DOI:** 10.3390/ijms262412032

**Published:** 2025-12-14

**Authors:** Vy Ho, Tommy Tran, Jaylan Patel, Betelhem Teshome, Vikrant Rai

**Affiliations:** 1 College of Osteopathic Medicine of the Pacific, Western University of Health Sciences, Pomona, CA 91766, USA; vy.ho@westernu.edu (V.H.); tommy.tran@westernu.edu (T.T.); jaylan.patel@westernu.edu (J.P.); 2 Department of Translational Research, Western University of Health Sciences, Pomona, CA 91766, USA

**Keywords:** wound healing, diabetes, CD36, CD69, inflammation

## Abstract

Diabetic foot ulcers (DFUs) are a leading cause of morbidity worldwide along with the risk of other chronic health issues. Hyperglycemia has been shown to alter immune regulation at the wound site and potentially contributes to non-healing DFUs. However, the effects of hyperglycemia on the expression of mediators of inflammation and angiogenesis (CD36), immune regulation (TLR-7 and CD69), and inflammation/tissue repair (CD274) regarding wound healing are unknown. This study aims to investigate the effects of hyperglycemia on the gene and protein expression of CD36, CD69, CD274, and TLR-7 during wound healing using a rat model of induced diabetes. The expression of these mediators was examined using cutaneous tissues from rat models of diabetes with cutaneous wounds. Skin samples *(n* = 7 each, control and healed) from the control and diabetic rats were analyzed using hematoxylin and eosin as well as trichrome staining, immunohistochemistry, and PCR. In vitro studies were conducted using rat fibroblasts. Hyperglycemia significantly increases the expression of CD36, CD69, and CD274 and increases TLR-7 expression but not significantly in diabetic tissues compared to control tissues. In vitro studies corroborate the findings in tissues. The modulation of these mediators by hyperglycemia suggests their probable role in delayed wound healing, and these mediators may be potential therapeutic targets to promote wound healing in DFUs.

## 1. Introduction

Diabetes, characterized by persistent hyperglycemia, increases the risk of myocardial infarction, stroke, microvascular events, and mortality. These risks are all strongly associated with hyperglycemia [1]. In addition, skin complications such as foot ulcers and xerosis are serious issues for diabetic patients. Microbial infections, chronic inflammation, and, eventually, limb/toe amputations are complications associated with non-healing ulcers [2]. The multifactorial pathogenesis of ischemia, infection, or inflammation of the diabetic and chronic wound environment interferes with the healing process, making it difficult to heal by releasing local factors that may worsen the wound [3].

The gold standard in healing and treating diabetic foot ulcers (DFUs) includes wound debridement, off-loading, antibiotics, wound dressing, and maintaining a clean wound environment. However, non-healing DFUs are still a critical problem with costly and prolonged treatment. Recurrence, amputation, and eventually death in some cases are common complications associated with non-healing DFUs. Factors including vasculopathy, decreased angiogenesis, ischemia, chronic inflammation, and wound environment interfere with the effect of endogenous factors. In a chronic inflammatory state, this results in a wound that remains in the inflammatory phase without progressing to the resolution phase [4,5,6]. The presence of hyperglycemia, dysregulated recruitment of neutrophils and macrophages, increased expression of proinflammatory cytokines including interleukin (IL)-1β, IL-6, tumor necrosis factor (TNF)-α, and IL-8, matrix metalloproteinases (MMPs), and serine proteases in the wound contribute to chronic inflammation and non-healing [4,7]. This study aims to investigate the effects of hyperglycemia on the expression of various factors, including surface receptors CD36, Toll-like receptor (TLR)-7, programmed death-ligand 1 (PD-L1, also known as CD274), and CD69, in tissues from diabetic rats compared to control rats.

We choose these factors because of their role in inflammation, angiogenesis, and extracellular matrix (ECM) remodeling, which are the pathophysiologies underlying non-healing DFUs. The expression of these mediators was increased in DFU tissues compared to control normal tissues in our RNA seq analysis (https://submit.ncbi.nlm.nih.gov/subs/sra/SUB14735487/, accessed on 28 October 2025). CD36, also known as platelet glycoprotein 4, is a multifaceted receptor on the surface of macrophages, monocytes, dendritic cells, T and B cells, and endothelial cells. CD36 is involved in inflammation, metabolism, and immunity, and it plays a role in atherosclerosis, diabetes, obesity, and certain cancers [8,9,10]. CD36′s role is context-dependent and may have a varied role in different types of cells. In endothelial cells, as a receptor for thrombospondin 1 (TSP1), CD36 attenuates angiogenesis promoted by growth factors, such as fibroblast growth factor 2 (FGF2) and vascular endothelial growth factor (VEGF). In macrophages and monocytes, CD36 promotes inflammatory responses, phagocytosis, and the uptake of bioactive lipids [8]. CD36 binds with ECM components like collagen and thrombospondin 1 (TSP1) and regulates the migration of immune cells. CD36 deficiency leads to altered ECM remodeling, including the abnormal deposition of fibronectin and collagen, and it contributes to tissue inflammation and impaired barrier function [11,12]. However, the role of CD36 in cutaneous wound healing and the effect of hyperglycemia on its expression are yet unknown.

CD69 is a protein and an early marker of immune cell activation, appearing on the surface of activated T lymphocytes, natural killer (NK) cells, and platelets within hours of stimulation. It is involved in immune response, including immune function, cell proliferation, signal transmission, and cell retention within tissues. The aberrant expression of CD69 has been linked to chronic inflammatory diseases [13,14,15]. CD69 may significantly influence ECM remodeling by controlling the inflammatory and fibrotic processes. By controlling inflammation and TGF-β, CD69 indirectly influences the extracellular matrix (ECM) remodeling, as inflammation and fibrosis are deeply intertwined processes involving ECM breakdown and buildup. It does this by affecting the production of cytokines like transforming growth factor (TGF)-β and IL-17 and by modulating the behavior of immune cells. It should also be noted that the engagement of CD69 on activated immune cells triggers the synthesis of active TGF-β1, which is a potent anti-inflammatory and anti-autoimmune cytokine. This localized production of TGF-β at inflammatory sites helps to downregulate the immune response, thereby preventing damage to the tissue [16,17]. These two studies have evaluated the role of CD69 in the intestine, but the role in cutaneous healing is largely unknown.

TLR-7, an innate immune receptor, plays a complex role in wound healing, primarily by triggering inflammation to fight infection and promote initial repair, but excessive or prolonged activation can lead to delayed healing and scarring. In the early stages, TLR-7 on cells like plasmacytoid dendritic cells (pDCs) can sense damage and release type I interferons, which are critical for early inflammatory responses and re-epithelialization. However, in chronic conditions like diabetes, elevated TLR-7 expression is linked to impaired healing, and stimulating TLR-7 with agonists has been shown to delay healing by prolonging inflammation, as seen in models of chronic wounds [18,19,20]. TLR-7 has a context-dependent role in angiogenesis and ECM remodeling. It may promote or inhibit angiogenesis. The activation of TLR-7 in macrophages can trigger an “angiogenic switch” by upregulating pro-angiogenic factors like VEGF [21]. In conditions like atherosclerosis, TLR-7 can have protective effects by limiting inflammation and stabilizing plaque, which influences ECM composition and stability. However, TLR-7 can also promote inflammation-driven tissue damage and pathological remodeling, such as post-heart attack scarring, through pathways like nuclear factor kappa beta (NF-κB) and other pro-fibrotic signals [22,23].

CD274 plays a positive and dynamic role in wound healing by resolving excessive inflammation. By binding to its receptor, programmed death 1 (PD-1), on immune cells, CD274 promotes the transition from the inflammatory phase to the proliferative phase, which prevents chronic inflammation and accelerates tissue repair. CD274 is also expressed by fibroblast-like cells in the wound’s granulation tissue during the inflammatory and early proliferative stages, forming a localized immunosuppressive environment that promotes inflammation resolution. CD274 also promotes M2 polarization [24]. This suggests that a persistently increased expression of CD36 and CD69 may be detrimental, and a persistently increased expression of TLR-7 and CD274 may be beneficial for wound healing. Based on this background, in this study, we investigated the effects of hyperglycemia on the expression of these mediators in the normal and healed skin tissues in a rat model of diabetes.

## 2. Results

### 2.1. Hyperglycemia Decreases Collagen Content in Healed Tissues

**Figure 1:**
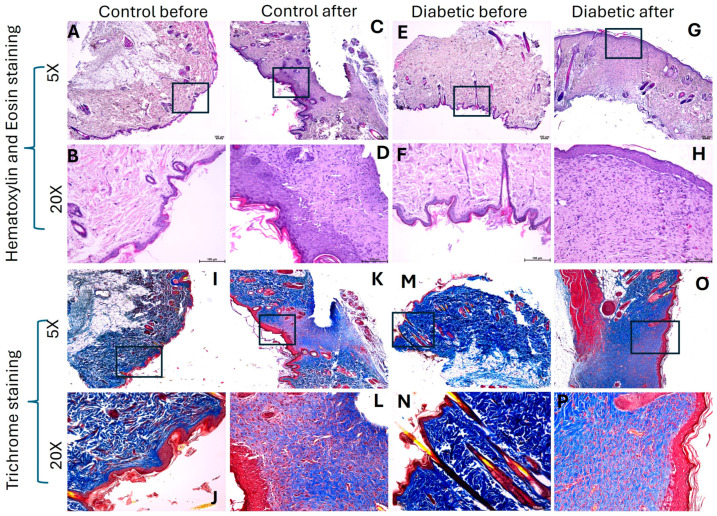
Hematoxylin and eosin and trichrome staining in control and healed tissues. H and E staining for control before (skin during wounding, panels (**A**,**B**,**E**,**F**)) and control after (skin collected after wound healed, panels (**C**,**D**,**G**,**H**)) in control and diabetic rats. Trichrome staining for control before (skin during wounding, panels (**I**,**J**,**M**,**N**)) and control after (skin collected after wound healed, panels (**K**,**L**,**O**,**P**)) in control and diabetic rats. Square/rectangle area in the 5× image shows area scanned at 20× with a scale of 100 μm.

In both control and diabetic rats, H and E staining revealed wound healing with scar tissue formation as well as a distorted architecture of healed tissues (Figure 1C,G). Healed tissues showed no sign of inflammation and were devoid of sweat glands, sebaceous glands, and hair follicles. Diabetic healed skin showed decreased collagen (blue color) compared to control, control healed, and diabetic skin in trichrome staining (Figure 1J,L,N,P). Using collagen staining on a scale of 0–5, with 0 as no color and 5 as deep blue color, diabetic healed skin showed 1, control healed skin showed 1–2, and control and diabetic skin showed 2–3, suggesting decreased collagens in diabetic healed tissues (Figure 1).

### 2.2. Hyperglycemia Modulates the Gene Expression of CD36, CD69, CD274, and TLR-7 in Healed Tissues

**Figure 2:**
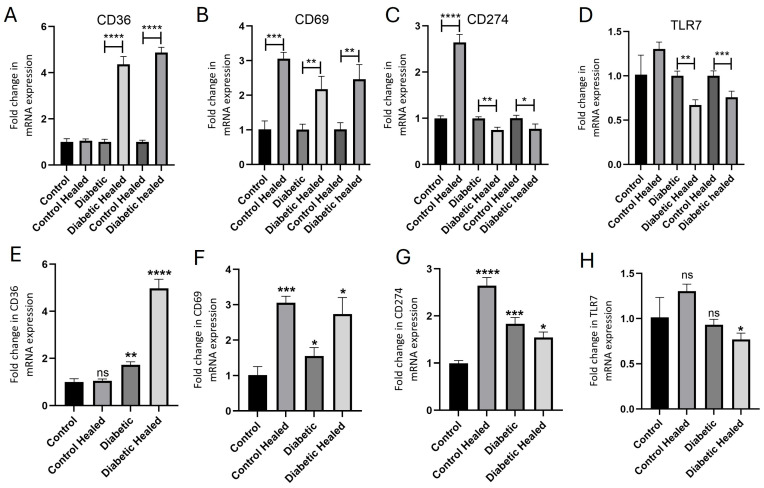
Real-time polymerase chain reaction (RT-PCR) for gene expression of *CD36*, *CD69*, *CD274*, and *TLR-7*. Panels (**A**–**D**) represent within-group comparison (control skin vs. control healed, diabetic skin vs. diabetic healed, and control healed vs. diabetic healed), while panels (**E**–**H**) represent gene expression compared to control. Data are presented as mean ± SD. * *p* < 0.05, ** *p* < 0.01, *** *p* < 0.001, and **** *p* < 0.0001.

RT-PCR studies revealed a significant increase in gene expression of *CD36* (*p* < 0.0001) and CD69 (*p* < 0.007), while there was a significant decrease in *CD274* (*p* < 0.002) and TLR-7 (*p* < 0.002) in diabetic healed tissues compared to control diabetic skin (Figure 2A–D). In the control skin and control healed skin, *CD36* expression was comparable, CD69 was significantly increased (*p* < 0.0002), *CD274* was significantly increased (*p* < 0.0001), and TLR-7 was minimally increased (Figure 2A–D). While comparing control and diabetic healed skin (Figure 2A–D), *CD36* and *CD69* expression were significantly increased (*p* < 0.0001 and *p* < 0.005, respectively), while *CD274* and *TLR-7* expression were significantly decreased (*p* = 0.032 and *p* < 0.0009, respectively). The comparison of all tissues compared to control skin revealed a significant increase in *CD36* expression (Figure 2E) in diabetic control (*p* = 0.0023) and healed skin (*p* = 0.00016), a significant increase in *CD69* (Figure 2F, *p* = 0.00029, 0.05, and 0.017, respectively) and *CD274* expression (Figure 2G, *p* < 0.0001, *p* = 0.0005, and 0.046, respectively) in control healed, diabetic control, and diabetic healed, and a significant decrease in the *TLR-7* expression of diabetic healed tissue (Figure 2H, *p* = 0.039).

### 2.3. Hyperglycemia Modulate the Protein Expression of CD36, CD69, CD274, and TLR-7 in Healed Tissues

**Figure 3:**
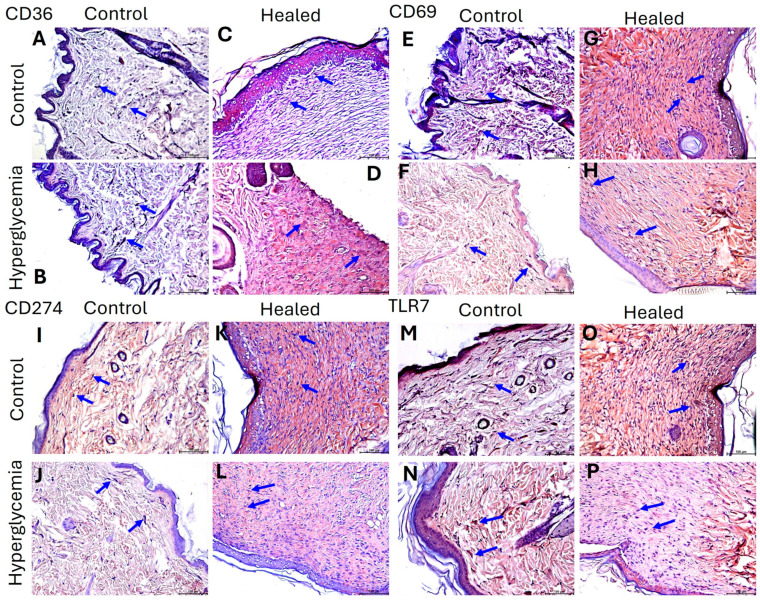
Immunohistochemistry staining for CD36, CD69, CD274, and TLR-7 in control (skin collected during wounding) and healed (skin collected after wound healed) skin in the control and diabetic rats. Panels (**A**–**D**) (CD36), (**E**–**H**) (CD69), (**I**–**L**) (CD274), and (**M**–**P**) (TLR-7). Control skin collected during wounding (panels (**A**,**C**,**E**,**G**,**I**,**K**,**M**,**N**)) and skin collected after sacrifice once the wound healed (panels (**B**,**D**,**F**,**H**,**J**,**L**,**N**,**P**)). All images were scanned at 20× with a scale of 100 μm. The blue arrow shows positive staining.

Immunohistochemistry (IHC) revealed an increased protein expression of CD36 in control healed and diabetic healed tissues compared to control (Figure 3A–D). CD36 expression in diabetic healed was greater than that in control healed (Figure 3C,D). CD36 expression was localized as well as diffused in both healed tissues, whereas it was mostly localized in control tissues. The protein expression of CD69 followed the same pattern except that the protein expression was higher in control healed tissues compared to diabetic healed tissues (Figure 3E–H). The protein expression for CD274 (Figure 3I–L) and TLR-7 (Figure 3M–P) showed greater expression in control healed tissues compared to diabetic healed tissues. The expression of CD69, CD274, and TLR-7 in control and diabetic control tissues was less than that in healed tissues and was localized compared to diffuse in healed tissues.

### 2.4. Hyperglycemia Modulate the Protein Expression of CD36, CD69, CD274, and TLR-7 in Fibroblasts

**Figure 4:**
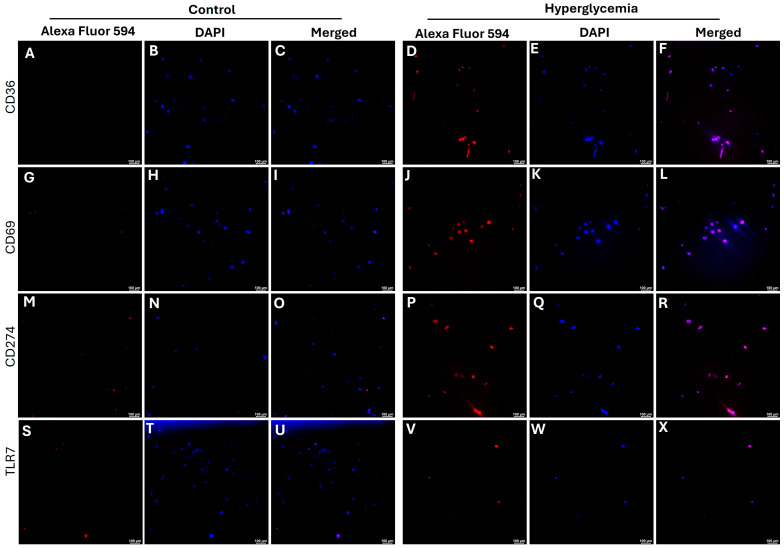
Immunofluorescence staining for CD36, CD69, CD274, and TLR-7 in fibroblasts cultured in control and 9% dextrose (hyperglycemic) medium. CD36 control (panels (**A**–**C**)) and hyperglycemic (panels (**D**–**F**)), CD69 control (panels (**G**–**I**)) and hyperglycemic (panels (**J**–**L**)), CD274 control (panels (**M**–**O**)) and hyperglycemic (panels (**P**–**R**)), TLR-7 control (panels (**S**–**U**)) and hyperglycemic (panels (**V**–**X**)). All images were scanned at 100μm.

The protein expression of CD36 (Figure 4D), CD69 (Figure 4J), and CD274 (Figure 4P) was increased with hyperglycemia compared to control cells (Figure 4A,G,M) as evidenced by increased fluorescence. There was no significant change in the protein expression of TLR-7 protein expression with hyperglycemia (Figure 4S–X).

### 2.5. Hyperglycemia Modulates the Gene Expression of CD36, CD69, and CD274 in Rat Fibroblasts

**Figure 5:**
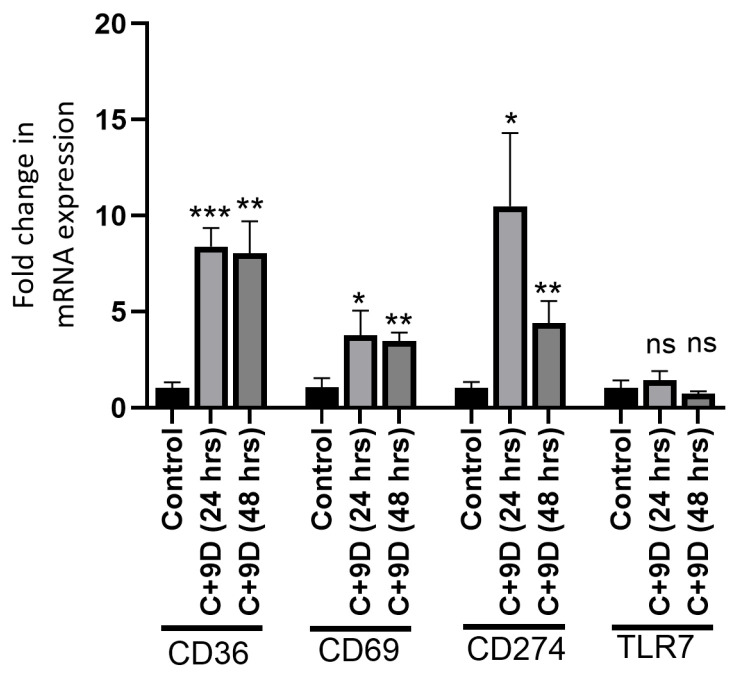
Real-time polymerase chain reaction (RT-PCR) for mRNA expression of CD36, CD69, CD274, and TLR-7 in fibroblasts. Data are presented as mean ± SD. * *p* < 0.05, ** *p* < 0.01, *** *p* < 0.001.

Hyperglycemia significantly increased the expression of CD36 (*p* = 0.00023 and *p* = 0.002), CD69 (*p* = 0.025 and *p* = 0.003), CD274 (*p* = 0.013 and *p* = 0.008), and TLR-7 (*p* = 0.36 and *p* = 0.23) at 24 and 48 h, but there was no significant effect on TLR-7 expression, and TLR-7 expression decreased at 48 h (Figure 5).

## 3. Discussion

Histological results revealed scar tissue in the healed skin. The healed tissues were devoid of hair cells, sebaceous glands, and sweat glands. No inflammation was noticed in the healed tissues. Compared to control tissues, healed tissues revealed the decreased collagen content in both control healed and diabetic healed tissues. Compared to controlled healed tissues, the diabetic healed tissues showed decreased collagen. These findings are supported by the fact that collagens decrease in diabetic healed tissues. This decrease is due to impaired production, altered remodeling, and increased degradation, which weakens the healed tissue. High glucose levels inhibit fibroblast proliferation, disrupt the normal cross-linking process, and lead to the accumulation of advanced glycation end-products (AGEs), which makes the collagen more fragile and susceptible to breakdown. This results in inferior mechanical properties, making diabetic wounds prone to chronic healing and complications [25,26]. Further, inflammation associated with diabetes can trigger collagen degradation and collagen structure damage, causing it to deteriorate. In some cases, collagen fragments can also act as mediators that attract immune cells and worsen inflammation [27,28]. It should be noted that the histological images do not reveal any focus of inflammation. This suggests that the healed wound had no significant infiltration of immune cells, but inflammation at the molecular level affects the process of wound healing which should be further investigated.

The effect of hyperglycemia on the expressions of CD36 and CD69 is supported by the significantly increased mRNA expression and protein expression of CD36 and CD69 in healed wounds in diabetic rats. High levels of CD36 are linked to increased inflammation and impaired insulin signaling in conditions like obesity and type 2 diabetes. CD36 promotes the accumulation of lipids and activation of immune cells, such as macrophages, which contributes to insulin resistance and β-cell dysfunction [29,30]. Although CD36 promotes inflammation, it is also needed for wound healing. CD36 is involved in wound healing by influencing inflammation, angiogenesis, and clearing cellular debris. It plays a dual role: it helps clear dead cells (efferocytosis), which is crucial for proper healing, but its anti-angiogenic function can hinder tissue repair. CD36 also contributes to the formation of fibrotic scars, which can be detrimental to wound healing [8,10,11,31,32]. The scarred tissue in the healed wound may be due to increased CD36. The change in the expression of CD36 with hyperglycemia may be due to regulation at the post-transcriptional and post-translational levels. High glucose concentrations enhance the efficiency of *CD36* mRNA translation into protein, leading to increased levels of the CD36 protein on the cell surface. For example, in atherosclerotic patients with hyperglycemia, there is an increase in post-translational modifications, specifically glycosylation, that contribute to higher functional CD36 levels. There is also evidence suggesting an increased recycling of CD36 to the cell surface. In cardiac injury related to diabetes, a positive feedback loop exists where high glucose rapidly induces CD36 protein, which in turn promotes further CD36 expression via microRNA-320 (miR-320) pathways [33,34,35].

CD69 is a marker for early immune cell activation and plays a complex and sometimes paradoxical role in inflammation and diabetes. CD69 functions as an immunoregulatory molecule that is altered in diabetic conditions, contributing to both inflammation and disease progression. CD69 is involved in different leukocyte subsets activation and the pathogenesis of chronic inflammation [14,36,37]. Early activation of immune cells can protect against immune damage after injury by suppressing inflammation, but high CD69 expression may also result in a weaker immune response, potentially hindering healing. The significant increase in *CD69* mRNA in both control and diabetic healed tissues suggests its role in immune regulation, but its increased protein expression in control healed tissues compared to diabetic control tissues suggests that CD69 expression may be impaired during diabetes. Immunofluorescence studies showed that hyperglycemia increases CD69 expression, but a comparative decrease in diabetic tissues compared to control tissues is an interesting finding. This may be due to the cells expressing CD69. CD69 is expressed by a variety of immune cells, including activated T and B lymphocytes, natural killer (NK) cells, neutrophils, eosinophils, and monocytes. It is also constitutively expressed on platelets and immature thymocytes, and it can be found on other cells like epidermal Langerhans cells and certain myeloid precursors [38]. The expression of CD69 is known to be influenced by the metabolic composition of the surrounding tissue environment. CD69 itself functions as a “metabolic gatekeeper” and regulates the uptake of amino acids and key signaling pathways like mTOR, which are central to T-cell function and activation [14]. A decreased immune response in diabetes may have led to these differences in cells and tissues. This aspect warrants further investigation.

Another interesting finding was the decreased expression of CD274 and TLR-7 in diabetic and control healed tissues. CD274 plays a crucial role in wound healing by modulating the inflammatory response and promoting tissue repair. CD274 is expressed by various cells involved in wound repair, including fibroblasts and keratinocytes, and promotes healing by resolving inflammation and facilitating cell migration [24,39]. As mentioned above, CD274 plays a complex role in inflammation by acting as a negative regulator of immune responses to minimize tissue damage, but cancer cells also use it to evade immune attack. An increased expression of CD274 in healed tissues may be due to increased immune response aiding in healing response to promote healing, and a decrease in the population of fibroblasts and keratinocytes in diabetic healed wounds may have resulted in decreased CD274 expression. Immunofluorescence showed an increased expression of CD274 with hyperglycemia, and this is supported by the fact that hyperglycemia can increase the expression of CD274 (PD-L1), often by altering cellular metabolism and activating specific signaling pathways, including epidermal growth factor receptor (EGFR), which activates downstream pathways like Ras-Extracellular signal-regulated kinase (RAS/ERK) [40]. It should be noted that immunofluorescence studies were conducted using fibroblasts; however, the tissue expression may be a mix of expression by various cells expression: CD274, including immune cells like activated T cells, B cells, monocytes, macrophages, and dendritic cells (https://www.ncbi.nlm.nih.gov/gene/60533; accessed on 28 October 2025). Increased expression in fibroblasts with hyperglycemia is supported by the fact that fibroblasts may express CD274 in certain conditions [41]. High glucose levels can stimulate the epidermal growth factor receptor (EGFR) and activate the RAS signaling pathway in some cancer cells, such as pancreatic tumor cells. This activation leads to a downregulation of an RNA-binding protein called PTRH1. The decrease in PTRH1, in turn, enhances the stability of *PD-L1* mRNA, leading to increased protein expression. Insulin can directly induce PD-L1 expression in pancreatic cancer cells via the insulin receptor-A and/or IGF-receptor-induced MAPK/ERK signaling pathway. Hyperglycemia contributes to an increased production of reactive oxygen species (ROS) or superoxides, leading to oxidative stress. Oxidative stress influences various signaling pathways, potentially impacting gene expression, including that of CD274 [42,43]. The mechanistic effects of hyperglycemia on CD274 expression have been investigated in other diseases, mainly oncoloy, but not in healing wounds.

TLR-7 expressions (both gene and protein) were decreased in diabetic control and healed tissues compared to control tissues. TLR-7 can promote healing by triggering initial immune responses to host-derived nucleic acids in wounds, but overactivation can cause prolonged inflammation and delayed healing. Studies show that while appropriately activating TLR-7 can aid healing, its overstimulation by agonists like resiquimod can hinder the process by causing excessive, sustained inflammation [44]. This notion is supported by an increased TLR-7 in control tissues in which the wound was healed in 14 days—compare to diabetics, where the wound healed in 21 days. TLR-7 can recognize host-derived nucleic acids released from damaged cells in a wound. This recognition activates nearby plasmacytoid dendritic cells (pDCs) to produce type I interferons (IFNs), which are crucial for the early inflammatory phase and help with the re-epithelialization of the skin [18]. The protein expression of TLR-7 in control and diabetic skin was comparable, while it was increased in control healed and decreased in diabetic healed tissues. Immunofluorescence studies also suggest that hyperglycemia does not have a significant effect on TLR-7 expression in fibroblasts. This effect of hyperglycemia was contrary to previous reports that hyperglycemia increases TLR-7 expression. This may be due to the time of hyperglycemia, as we only incubated cells for 24 h for immunofluorescence. There was no significant change in the expression of TLR-7 even at 48 h. TLR-7 plays a dual role in wound healing, promoting an early inflammatory response crucial for initial repair, and a later pro-resolving and anti-angiogenic response that can prevent excessive scarring. After skin injury, TLR-7 is activated by host-derived nucleic acids, stimulating plasmacytoid dendritic cells (pDCs) to produce type I interferons (IFNs), which are vital for early inflammation and re-epithelialization. Conversely, later activation can lead to inflammation resolution and inhibit angiogenesis, while dysregulated activation in chronic wounds can contribute to prolonged inflammation and poor healing [18,45,46]. Mechanistically, high glucose conditions activate key inflammatory signaling pathways, including protein kinase C (PKC), NADPH oxidase, and nuclear factor-κB (NF-κB). The activation of NF-κB, a transcription factor, promotes the expression of downstream genes, including various TLRs (such as TLR2, TLR4, and TLR-7) [47].

**Figure 6:**
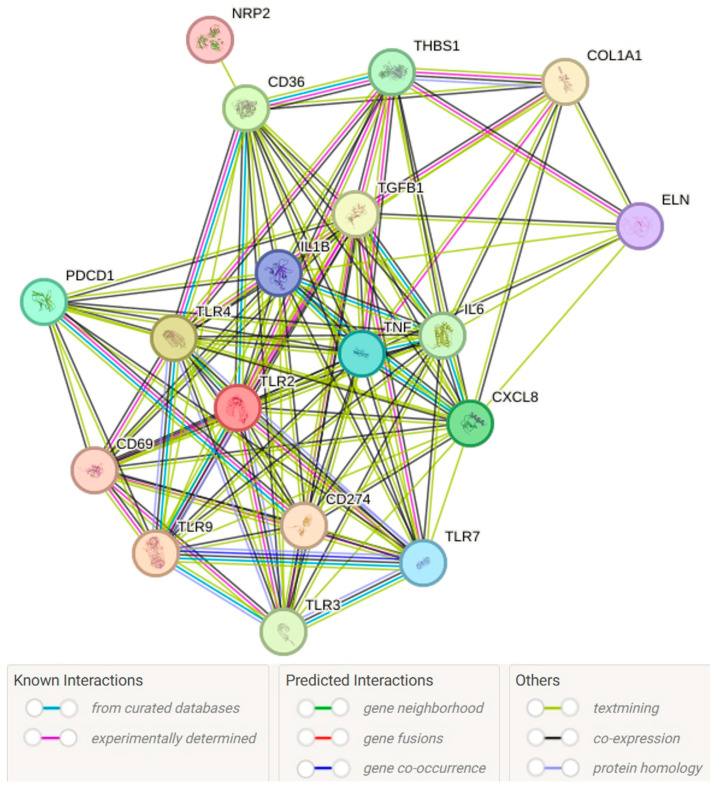
STRING network analysis depicting the interaction between CD36, CD69, CD274, and TLR-7 with mediators of inflammation, angiogenesis, and extracellular matrix remodeling.

As discussed above, the regulation of various cytokines, TGF-β, proteases, and collagens by CD36, CD69, CD274, and TLR-7 points toward the notion that these mediators may regulate wound healing. This notion is further supported by the interactive network analysis using STRING (assessed on 9 December 2025) showing interaction between these factors with mediators regulating inflammation, angiogenesis and ECM remodeling (Figure 6). As shown in Figure 6, COL1A1, TGFB2, and ELN involve ECM remodeling, THBS1 suppresses angiogenesis, CXCL8 in low concentrations promotes angiogenesis while its higher expression attenuates angiogenesis, and IL6, IL1B, TLR2, TLR4, TLR3, and TNF are involved in the regulation of inflammation. The interaction of CD36, CD69, CD274, and TLR-7 with these mediators supports their role in wound healing.

Although the exact role of these mediators in wound healing is not completely understood, the altered expression of CD36, CD69, CD274, and TLR-7 during wound healing suggests that targeting these mediators may have therapeutic potential in promoting wound healing. This is because CD36 is involved in regulating inflammation, immune response, angiogenesis, and ECM remodeling; CD69 regulates immune response, inflammation, and ECM remodeling; CD274 plays a role in regulating wound healing and inflammation; while TLR-7 regulates immune response, wound healing, angiogenesis, and inflammation.

## 4. Materials and Methods

### 4.1. Animal Model

This study used tissues already collected from the diabetic rat model with wound healing developed in the lab. We used 8–10-week-old male and female Sprague–Dawley rats purchased from Charles River Laboratories, Wilmington, MA, USA. Rats were maintained at the Western University Animal Resource facility per the Standard Operating Procedure with a 12:12 h light–dark cycle at constant temperature (22 °C) and under the supervision of the Manager, Animal Facility, and the attending veterinarian. The animal facility features a sentinel program as well as controlled lighting, air circulation, air conditioning, and drainage systems. All procedures involving the use and care of animals were conducted in accordance with the guidelines approved by WesternU IACUC (R24IACUC013). Normal skin tissues (during wounding, hereafter control tissues) and healed tissues (after sacrifice) were collected from nondiabetic (control) and diabetic rats (*n* = 7 each, a total of 28). The control rats were fed with a normal diet (ND; 20% protein, 70% carbohydrate, 10% fat; D12450B; Research Diet Inc. New Brunswick, NJ, USA) and diabetic rats were fed with a high-fat diet (HFD; 45% fructose (carbohydrate), 5% protein, 45% fat; 5.7 Kcal/g total; #D12451; Research Diet Inc. New Brunswick, NJ, USA) with water *ad libitum*. Diabetes was induced with a low-dose (25 mg/kg, dissolved in 0.1 M sodium citrate buffer at pH 4.4) streptozotocin (D-isomer- the active form, STZ; Sigma-Aldrich, St. Louis, MO, USA) injected intraperitoneally after 6 weeks of HFD. Blood glucose levels were monitored in both groups by tail vein blood using an AlphaTrak glucometer (ThermoFisher, Waltham, MA, USA). Rats with a blood glucose level greater than 300 mg/dL for a minimum of two weeks after were considered diabetic. The tissues were collected in 10% formalin for histology and in RNA later for RNA extraction.

### 4.2. Tissue Processing

The tissues in formalin were processed in LEICA ASP6025 Tissue Processor, which was followed by paraffin embedding and sectioning using a Leica microtome. Then, the slides were baked for one hour at 60 °C, and the temperature should not be higher than 60 °C because higher temperatures may damage delicate tissues.

### 4.3. Histology

For this project, hematoxylin and eosin (H&E) staining and trichrome staining were performed to examine the tissue architecture and collagen staining, respectively. For all the staining, the solutions need to be fresh to avoid contamination or cross-staining from other solutions. For H&E staining, the slides were deparaffinized and rehydrated using xylene and varying concentrations of ethanol (100% EtOH for 5 min, 95% EtOH for 2 min, 80% EtOH for 2 min, 70% EtOH for 2 min). Then, the slides were washed with DI water for 5 min, which was followed by staining with hematoxylin (8–10 dips) and washing under running tap water for 3–5 min until the water was clear. The slides were dipped in bluing solution 10 times and then were stained with eosin solution for 2–3 dips. Again, the slides were washed in a series of decreasing concentrations of ethanol, air dried at room temperature, and mounted with CytoSeal 60 and slide covers.

To perform trichrome staining, after deparaffinization and dehydration, tissues were refixed in Bouin’s solution for 1 h at 56 °C to improve staining quality. Then, the slides were rinsed under running tap water for 5–10 min to remove the yellow color, after which the slides were stained in Weigert’s iron hematoxylin (1:1 ratio mix A and B) working solution for 10 min. Afterwards, the slides were rinsed in warm tap water for 10 min and washed in distilled water for 2–3 min. Next, the slides were stained in Bierbrich scarlet acid fuchsin solution for 6 min, which was followed by washing in distilled water for 2–3 min. Then, slides were differentiated in phosphomolybdic-phosphotungstic acid solution for 10 min, transferred directly without rinsing to aniline blue solution, stained for 6–7 min, and then transferred to 1% acetic acid solution for 2 min twice. The slides were then washed in distilled water. Then, they were rehydrated very quickly through 95% EtOH for 5 dips, 100% EtOH for five dips, and Xylene for 2 min. Finally, the slides were air-dried and mounted with CytoSeal 60 and cover slips.

### 4.4. Immunohistochemistry

For immunohistochemistry, the slides were deparaffinized and dehydrated using xylene and ethanol. Antigen retrieval was performed by placing the slides in 1% citrate buffer and steaming them for 15 min. The slides were removed and then cooled down to room temperature. The slides were then washed in PBS for 5 min, and the tissues were circled with a hydrophobic PAP Pen. The endogenous peroxidase activity was then neutralized with 3% hydrogen peroxide for 15 min and then washed in PBS twice for 5 min each. This was followed by blocking non-specific receptors for 1 h at room temperature. After tipping off the blocking solution, the slides were incubated with CD36 (NB400-144), CD69 (PA5-114989), CD274 (14-5982-82), and TLR-7 (NBP2-24906) primary antibodies overnight at 4 °C. The next day, the slides were washed with PBS twice for 5 min and incubated with a secondary antibody from Vetastain Kit (Vector labs, Newark, NJ, USA) for one hour. After washing with PBS, the tissues were incubated with ABC solution for 30 min and then washed with water. The AEC solution was used as a chromogen to detect antigen, and slides were washed after 2–3 min once the color was developed. The slides were then washed with water, counterstained with hematoxylin, washed, and mounted with CytoSeal60 and coverslips.

### 4.5. Quantitative Real-Time Polymerase Chain Reaction (RT-qPCR)

PCR was used to analyze the gene expression of CD36, CD69, CD274, and TLR-7 in rat skin tissues. The total RNA was prepared using TRIZOL (Sigma Aldrich T9424, St. Louis, MO, USA), quantified using Nanodrop 2000 (ThermoFisher, Waltham, MA, USA), and cDNA was prepared using an iScript cDNA synthesis kit (BioRad #1708891, Irvine, CA, USA) following the manufacturer’s instructions. RT-qPCR was conducted with the cycling of 5 min at 95 °C for initial denaturation, 40 cycles of the 30 s at 95 °C, 30 s at 55–60 °C (based on primer annealing temperatures), and 30 s at 72 °C followed by melting curve analysis in triplicate using SYBR Green Master Mix and a real-time PCR system (CFX96, BioRad Laboratories, and Hercules, CA, USA). After normalizing with the 18S housekeeping gene, the fold change in mRNA expression was calculated using the 2^−ΔΔCT^ method. The oligonucleotide primers used in RT-qPCR (Table 1) were purchased from Integrated DNA Technology (IDT Coralville, IA 52241, USA).

### 4.6. Cell Culture and In Vitro Studies

After culturing the rat fibroblasts to 90% confluence in Dulbecco’s Modified Eagle’s Medium (DMEM) with 10% fetal bovine serum and 1% penicillin streptomycin in a humidified incubator with 5% CO_2_ at 37 °C, the cells were trypsinized. First, 1 × 10^6^ cells were plated in each well of a 6-well plate for gene expression analysis and 8 × 10^3^ cells in each chamber of the slide for immunofluorescence. Cells were treated with hyperglycemic medium (DMEM with 9.0 g/L glucose) with proper control and were collected for RNA extraction at 24 h, 48 h, and 72 h for 24 h for immunofluorescence. The total RNA was extracted using TRIZOL, cDNA was prepared, and RT-qPCR was conducted for mRNA expression. For immunofluorescence, the cells were washed with sterile PBS after 24 h, fixed with 10% formaldehyde, and incubated in 0.01% Triton for 10 min. Then, cells were washed with PBS and incubated with blocking solution for one hour, which was followed by incubation with CD36, CD69, CD274, and TLR-7 primary antibodies overnight at 4 °C followed by washing with PBS and incubation with Alexa Fluor 594 for 30 min. The cells were washed, counterstained with DAPI, and scanned using a Leica Fluorescence microscope.

### 4.7. Statistical Analysis

All data are presented as mean ± SD. The data were analyzed using GraphPad Prism 10, and comparison between the two groups was analyzed using Student’s *t*-test for statistical significance. A probability value of < 0.05 was considered significant. * *p* < 0.05, ** *p* < 0.01, *** *p* < 0.001, and **** *p* < 0.0001.

## 5. Conclusions

In conclusion, the healed tissues showed a significant decrease in collagen content, which was likely due to the effects of elevated glucose levels and chronic inflammation. Furthermore, the healed tissues exhibited an increased expression of CD36 and CD69. CD36 plays a critical role in wound healing by promoting inflammation and contributing to the formation of fibrotic scars, whereas CD69 activates immune cells to protect against immune-mediated damage. Hyperglycemia also modulated the expression of CD274 and TLR-7. The involvement of CD36, CD69, CD274, and TLR-7 in inflammation, angiogenesis, and ECM remodeling, the three components necessary for wound healing, makes these mediators an attractive target to promote wound healing by attenuating inflammation and promoting angiogenesis and ECM remodeling.

Limitations of the study and future perspective: The study highlights the effects of hyperglycemia on the expression of CD36, CD69, CD274, and TLR-7 in rat tissues and rat fibroblasts. It was also evident that control tissues healed in 14 days, whereas diabetic tissues healed in 21 days. This suggests that the altered expression of these mediators may have contributed to delayed healing. This aspect should be investigated in more depth both in vitro and in vivo. This study used only fibroblasts; further studies should be conducted using keratinocytes, vascular smooth muscle cells, endothelial cells, and immune cells to observe the effects of hyperglycemia. Further, co-culture studies with and without hyperglycemia using cells involved in wound healing (fibroblasts and keratinocytes) with immune cells, mainly macrophages and neutrophils, to investigate the effects on the expression of these mediators should be conducted. The expression of these mediators is not always present on fibroblasts and increases with stimulation; the effects on expression should also be investigated after stimulating cells with lipopolysaccharides (a mediator of inflammation).

## Figures and Tables

**Table 1:** Forward and reverse primer sequences used in RT-qPCR.

Gene	Primer Sequence (5′–3′)
CD36	Forward: GAAGCCAATCTTACGGTCCTG Reverse: CACATTTCAGAAGGCGGCAA
CD69	Forward: TAGACTGCGAGGCAAACTTAC Reverse: GCTATGGCACAGTCACCTATAC
CD274	Forward: TTACAGGTTGTCCCTGGTAATG Reverse: CCTCCAGGAAACAGTGTCTATG
TLR-7	Forward: TCCTTGAGTGGCCTACAAATC Reverse: CTTCAGAGAGCTAGACTGTTTCC
18S	Forward: GTAACCCGTTGAACCCCATT Reverse: CCATCCAATCGGTAGTAGCG

## Data Availability

All data related to this manuscript is included.

## Abbreviations

The following abbreviations are used in this manuscript:

AGEs	Advanced glycation end-products
DFUs	Diabetic foot ulcers
ECM	Extracellular matrix
IL	Interleukin
MMPs	Matrix metalloproteinases
NF-κB	Nuclear factor kappa beta
PD-L1	Programmed Cell Death Ligand 1
TNF-α	Tumor necrosis factor alpha
TLR-7	Toll-like receptor-7
TSP1	Thrombospondin 1
TGF-β	Transforming growth factor beta
VEGF	Vascular endothelial growth factor
